# Genome-Wide Association Study and Gene Expression Analysis Identifies *CD84* as a Predictor of Response to Etanercept Therapy in Rheumatoid Arthritis

**DOI:** 10.1371/journal.pgen.1003394

**Published:** 2013-03-28

**Authors:** Jing Cui, Eli A. Stahl, Saedis Saevarsdottir, Corinne Miceli, Dorothee Diogo, Gosia Trynka, Towfique Raj, Maša Umiċeviċ Mirkov, Helena Canhao, Katsunori Ikari, Chikashi Terao, Yukinori Okada, Sara Wedrén, Johan Askling, Hisashi Yamanaka, Shigeki Momohara, Atsuo Taniguchi, Koichiro Ohmura, Fumihiko Matsuda, Tsuneyo Mimori, Namrata Gupta, Manik Kuchroo, Ann W. Morgan, John D. Isaacs, Anthony G. Wilson, Kimme L. Hyrich, Marieke Herenius, Marieke E. Doorenspleet, Paul-Peter Tak, J. Bart A. Crusius, Irene E. van der Horst-Bruinsma, Gert Jan Wolbink, Piet L. C. M. van Riel, Mart van de Laar, Henk-Jan Guchelaar, Nancy A. Shadick, Cornelia F. Allaart, Tom W. J. Huizinga, Rene E. M. Toes, Robert P. Kimberly, S. Louis Bridges, Lindsey A. Criswell, Larry W. Moreland, João Eurico Fonseca, Niek de Vries, Barbara E. Stranger, Philip L. De Jager, Soumya Raychaudhuri, Michael E. Weinblatt, Peter K. Gregersen, Xavier Mariette, Anne Barton, Leonid Padyukov, Marieke J. H. Coenen, Elizabeth W. Karlson, Robert M. Plenge

**Affiliations:** 1 Division of Rheumatology, Immunology, and Allergy, Brigham and Women's Hospital, Harvard Medical School, Boston, Massachusetts, United States of America; 2 Division of Genetics, Brigham and Women's Hospital, Harvard Medical School, Boston, Massachusetts, United States of America; 3 Medical and Population Genetics Program, Chemical Biology Program, Broad Institute, Cambridge, Massachusetts, United States of America; 4 Rheumatology Unit, Department of Medicine, Karolinska Institutet and Karolinska University Hospital Solna, Stockholm, Sweden; 5 Institute of Environmental Medicine, Karolinska Institutet, Stockholm, Sweden; 6 Université Paris-Sud, Orsay, France; 7 APHP–Hôpital Bicêtre, INSERM U1012, Le Kremlin Bicêtre, Paris, France; 8 Program in Translational NeuroPsychiatric Genomics, Institute for the Neurosciences, Department of Neurology, Brigham and Women's Hospital, Boston, Massachusetts, United States of America; 9 Department of Human Genetics, Radboud University Nijmegen Medical Centre, Nijmegen, The Netherlands; 10 Rheumatology Research Unit, Instituto de Medicina Molecular, Faculdade de Medicina da Universidade de Lisboa, Lisbon, Portugal; 11 Rheumatology Department, Santa Maria Hospital–CHLN, Lisbon, Portugal; 12 Institute of Rheumatology, Tokyo Women's Medical University, Tokyo, Japan; 13 Department of Rheumatology and Clinical Immunology, Kyoto University Graduate School of Medicine, Kyoto, Japan; 14 Center for Genomic Medicine, Kyoto University Graduate School of Medicine, Kyoto, Japan; 15 Clinical Epidemiology Unit, Department of Medicine, Karolinska Institute/Karolinska University Hospital, Stockholm, Sweden; 16 NIHR–Leeds Musculoskeletal Biomedical Research Unit and Leeds Institute of Molecular Medicine, University of Leeds, Leeds, United Kingdom; 17 Musculoskeletal Research Group, Institute of Cellular Medicine, Newcastle Upon Tyne, United Kingdom; 18 Rheumatology Unit, Medical School, University of Sheffield, Sheffield, United Kingdom; 19 School of Translational Medicine, Arthritis Research UK Epidemiology Unit, University of Manchester, Manchester, United Kingdom; 20 Department of Clinical Immunology and Rheumatology, Academic Medical Center/University of Amsterdam, Amsterdam, The Netherlands; 21 Laboratory of Immunogenetics, Department of Pathology, Vrije Universiteit Medical Center, Amsterdam, The Netherlands; 22 Department of Rheumatology, Vrije Universiteit University Medical Center, Amsterdam, The Netherlands; 23 Sanquin Research Landsteiner Laboratory, Academic Medical Center, University of Amsterdam, Amsterdam, The Netherlands; 24 School of Medicine and Biomedical Sciences, Sheffield University, Sheffield, United Kingdom; 25 Jan van Breemen Institute, Amsterdam, The Netherlands; 26 Arthritis Center Twente, University Twente and Medisch Spectrum Twente, Enschede, The Netherlands; 27 Department of Clinical Pharmacy and Toxicology, Leiden University Medical Center, Leiden, The Netherlands; 28 Department of Rheumatology, Leiden University Medical Centre, Leiden, The Netherlands; 29 Department of Medicine, University of Alabama at Birmingham, Birmingham, Alabama, United States of America; 30 Rosalind Russell Medical Research Center for Arthritis, Division of Rheumatology, Department of Medicine, University of California San Francisco, San Francisco, California, United States of America; 31 Division of Rheumatology and Clinical Immunology, University of Pittsburgh, Pittsburgh, Pennsylvania, United States of America; 32 NIHR Manchester Musculoskeletal Biomedical Research Unit, Central Manchester NHS Foundation Trust, Manchester Academic Health Sciences Centre, Manchester, United Kingdom; 33 The Feinstein Institute for Medical Research, North Shore–Long Island Jewish Health System, Manhasset, New York, United States of America; 34 Arthritis Research UK Epidemiology Unit, Musculoskeletal Research Group, University of Manchester, Manchester Academic Health Sciences Centre, Manchester, United Kingdom; North Carolina State University, United States of America

## Abstract

Anti-tumor necrosis factor alpha (anti-TNF) biologic therapy is a widely used treatment for rheumatoid arthritis (RA). It is unknown why some RA patients fail to respond adequately to anti-TNF therapy, which limits the development of clinical biomarkers to predict response or new drugs to target refractory cases. To understand the biological basis of response to anti-TNF therapy, we conducted a genome-wide association study (GWAS) meta-analysis of more than 2 million common variants in 2,706 RA patients from 13 different collections. Patients were treated with one of three anti-TNF medications: etanercept (n = 733), infliximab (n = 894), or adalimumab (n = 1,071). We identified a SNP (rs6427528) at the *1q23* locus that was associated with change in disease activity score (ΔDAS) in the etanercept subset of patients (*P* = 8×10^−8^), but not in the infliximab or adalimumab subsets (*P*>0.05). The SNP is predicted to disrupt transcription factor binding site motifs in the 3′ UTR of an immune-related gene, *CD84*, and the allele associated with better response to etanercept was associated with higher *CD84* gene expression in peripheral blood mononuclear cells (*P* = 1×10^−11^ in 228 non-RA patients and *P* = 0.004 in 132 RA patients). Consistent with the genetic findings, higher *CD84* gene expression correlated with lower cross-sectional DAS (*P* = 0.02, n = 210) and showed a non-significant trend for better ΔDAS in a subset of RA patients with gene expression data (n = 31, etanercept-treated). A small, multi-ethnic replication showed a non-significant trend towards an association among etanercept-treated RA patients of Portuguese ancestry (n = 139, *P* = 0.4), but no association among patients of Japanese ancestry (n = 151, *P* = 0.8). Our study demonstrates that an allele associated with response to etanercept therapy is also associated with *CD84* gene expression, and further that *CD84* expression correlates with disease activity. These findings support a model in which *CD84* genotypes and/or expression may serve as a useful biomarker for response to etanercept treatment in RA patients of European ancestry.

## Author Summary

There are no genetic predictors of response to one of the most widely used classes of drugs in the treatment of rheumatoid arthritis—biological modifiers of the inflammatory cytokine tumor necrosis factor-alpha (or anti-TNF therapy). To identify genetic predictors, we performed the largest genome-wide association study (GWAS) to date as part of an international collaboration. In our study, which included 2,706 RA patients treated with one of three anti-TNF drugs, the most significant finding was restricted to RA patients treated with etanercept (*P* = 8×10^−8^), a drug that acts as a soluble receptor to bind circulating cytokine and prevents TNF from binding to its cell surface receptor. The associated variant influences expression of a nearby immune-related gene, *CD84*, whose expression is correlated with disease activity in RA patients. Together, our data support a model in which genomic factors related to *CD84* expression serve as a predictor of disease activity and response to etanercept therapy among RA patients of European ancestry, but not anti-TNF therapies that act through different biological mechanisms or potentially in RA patients of other genetic ancestries.

## Introduction

Rheumatoid arthritis (RA) is an autoimmune disease characterized by chronic inflammation of the synovial lining of the joint [1]. If left untreated, outcome varies from self-limited disease in a small proportion of RA patients to severe disease resulting in profound structural damage, excess morbidity and disability, and early mortality [2]. In the last twenty years, disease activity has been controlled in many patients by treatment with disease-modifying anti-rheumatic drugs (DMARDs), such as methotrexate, and the more recently developed biologic DMARDs that block inflammatory cytokines such as tumor necrosis factor-alpha (TNFa) [3]. Unfortunately, these medications are not effective in all RA patients, with up to one-third of patients failing to respond to any single DMARD [1]–[3]. Moreover, the biological mechanisms underlying treatment failure are unknown, which limits the development of clinical biomarkers to guide DMARD therapy or the development of new drugs to target refractory cases.

There are two classes of anti-TNF therapy: the TNF receptor fusion protein (etanercept), which acts as a soluble receptor to bind circulating cytokine and prevent TNF from binding to its cell surface receptor, and monoclonal antibodies that bind TNF (adalimumab, infliximab, certolizumab, and golimumab). There are undoubtedly shared mechanisms between the two drug classes (e.g., downstream signaling factors), as illustrated by similar effects on the change in inflammatory cytokines, complement activation, lymphocyte trafficking, and apoptosis [4], [5], [6]. Similarly, there are likely to be different biological factors that influence response: infliximab and adalimumab are approved for treatment of Crohn's disease; infliximab and adalimumab bind to transmembrane TNF on the surface of activated immune cells, whereas etanercept only binds soluble TNF [7]; and etanercept also binds a related molecule, lymphotoxin alpha (LTA), whereas infliximab/adalimumab do not [8].

Pharmacogenetics of response to anti-TNF therapy in RA remains in its early stages, with no single variant reaching an unambiguous level of statistical significance. Candidate gene studies suggest associations of TNFa or TNF receptor alleles, RA risk alleles or other SNPs with response to anti-TNF therapy [9], [10], [11]. Two GWAS in small sample sets (largest was 566 patients) have been performed, which identified loci with suggestive evidence for association [12], [13]. Therefore, GWAS of large sample sizes may yet uncover genetic factors associated with response to anti-TNF therapy in RA, and larger cohorts enable separate analyses of the different types of anti-TNF drugs.

Here we report a GWAS of 2,706 samples with anti-TNF treatment response data collected from an international collaboration, including previously published GWAS data [12], [13]. Our primary outcome measure was the change in disease activity score based on a joint count in 28 joints (DAS28) from baseline to 3–12 months after initiating anti-TNF therapy. Our secondary outcome measure was European League Against Rheumatism (EULAR) responder status [14], [15], where patients are classified as EULAR good responders, moderate responders or non-responders based on follow up DAS28 after treatment and overall change in DAS28. We found a highly significant association for a variant that we also show is also a strong expression quantitative trait locus (eQTL) for the *CD84* gene. Our findings suggest that *CD84* genotype and/or expression may prove to be a biomarker for etanercept response in RA patients.

## Results

### Genome-wide association study

Clinical and GWAS data were compiled for 2,706 individuals of European ancestry from 13 collections as part of an international collaboration. Table 1 shows sample sizes, phenotypes and clinical variables for the four collections that were the units of analysis (additional details are shown in Table S1). Disease activity score based on a 28-joint count (DAS28) were collected at baseline and at one time point after anti-TNF therapy administration (mean 3.7 months, range 3–12 months). We defined our primary phenotype as a change in DAS28 (ΔDAS) from baseline (so that greater ΔDAS corresponded with better response to therapy; overall mean and standard deviation of 2.1±1.3), adjusted for baseline DAS. A secondary phenotype was used based on European League Against Rheumatism (EULAR) response criteria. EULAR ‘good response’ was defined as ending DAS<3.2 and ΔDAS>1.2; ‘non-response’ was defined as ΔDAS <0.6 or ΔDAS≤1.2, and ending DAS >5.1; and ‘moderate response’ is in between [15]. We limited our secondary analysis to a dichotomous outcome, EULAR good responders (n = 998 for all patients treated with anti-TNF therapy) versus EULAR non-responders (n = 655), excluding the moderate category based on the hypothesis that a more extreme phenotype of response would yield improved discrimination.

**Table 1:** Samples and clinical data.

Collection (analysis batch):	REF	BRAGGSS	DREAM	ReAct	Total
**Sample sizes**	959*	595	880*	272	2706
**Drug subsets**					
**etanercept**	365	259	109	0	733
**infliximab**	415	268	211	0	894
**adalimumab**	174	68	557	272	1071
**EULAR Reponse categories**					
**Good responder**	432**	161	313	92	998
**Moderate responder**	243	258	359	131	991
**Non-responder**	322	176	208	49	755
**Genotype platform**	mixed	Affy 500K	Illu550K +650K	Illumina OmniExpress	
**Clinical variables**					
**Age, yr; mean (SD)**	53.6 (12.7)	57.4 (10.9)	54.8 (12.9)	53.9 (10.8)	
**Disease duration, yr; mean (SD)**	6.7 (9.4)	14 (9.8)	9.6 (9.5)	12 (9.1)	
**Gender, female %**	75.6	77.3	68.3	77.9	
**Seropositive, %**	87	78	80	70	
**MTX co-therapy, %**	65.6	85.6	76.0	50.0	
**Baseline DAS, mean (SD)**	5.5 (1.2)	6.7 (0.9)	5.5 (1.2)	5.9 (1.0)	
**ΔDAS, mean (SD)**	1.9 (1.6)	2.5 (1.5)	1.9 (1.3)	2.2 (1.3)	
**Mean treatment duration**	4.6	5.6	3	3	
**Study design**	All***	Observational	Observational	Observational	

* 8 patients had no TNF drug information.

** 38 patients had only EULAR response (good, moderate or none) clinical data.

*** ABCoN, GENRA are prospective cohorts, BeSt, eRA and TEAR are randomized controlled trial (RCT), and rest of REF group are observational cohorts.

Clinical variables were examined for association with phenotype, and therefore possible confounding in genetic association tests. In multivariate models (Table S2), only baseline DAS was strongly associated with the ΔDAS phenotype. As previously shown [11], age and gender showed univariate associations that were attenuated in the multivariate analysis. Accordingly, we used only baseline DAS as a clinical covariate, as this allowed us to maximize sample size given clinical variable missing data in some cohorts.

We performed quality control (QC) filtering and data processing of GWAS data for each of eleven genotyping batches. Genotyping array platforms are described in the Methods. HapMap2 imputation allowed us to test for association at >2 M SNPs with imputation quality scores >0.5. Genotype data were merged across several genotype batches to create four collections for genome-wide association testing. We performed linear regression association tests using baseline DAS and three principal components as covariates, and performed inverse-variance weighted meta-analysis to combine results across the four collections. Quantile-quantile plots with genomic control *λ*_GC_ values are shown in Figure S1. We found no evidence of systematic inflation of association test results, and no evidence of deflation for imputed versus genotyped SNPs. As a final filter, we excluded SNPs that showed strong evidence of heterogeneity across collections (Cochran's Q P<0.001).

**Figure 1:**
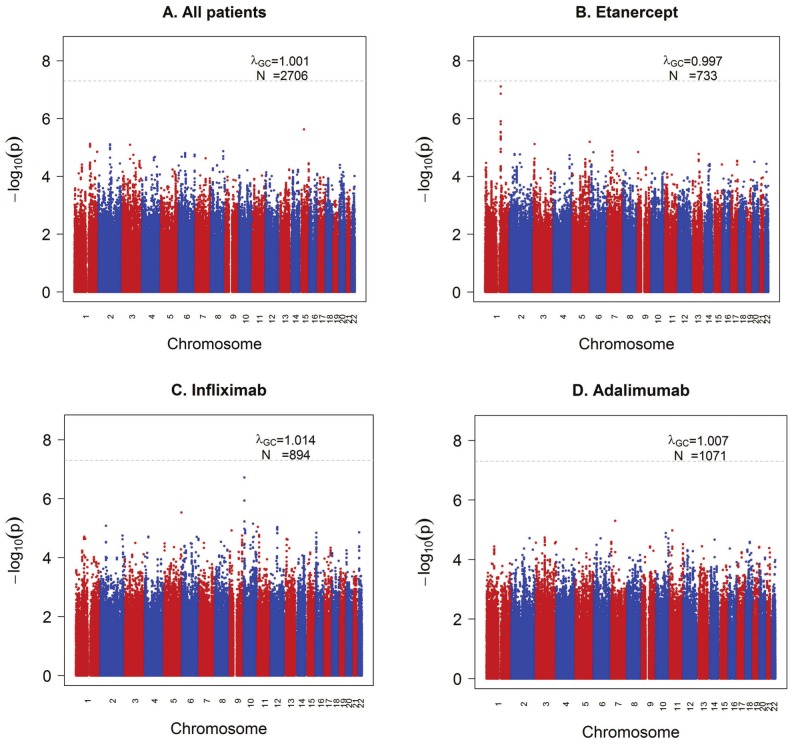
GWAS results for the ΔDAS phenotype. Shown are strengths of association (−Log10 P-value) for each SNP versus position along chromosomes 1 to 22. A) All samples (n = 2,706). B) Etanercept-treated patients (n = 733). C) Infliximab-treated patients (n = 894). D) Adalimumab-treated patients (n = 1,071).

We first analyzed all samples together (n = 2,706), regardless of drug type. We found no clear evidence of association with treatment response measured by ΔDAS (Figure 1A). Similar results were obtained using the binary phenotype of EULAR responder versus EULAR non-responder status ( Figures S1 and S2).

We next separately analyzed patients treated with either etanercept (n = 733), infliximab (n = 894) or adalimumab (n = 1,071) (Figure 1B–1D), under the hypothesis that different genetic loci affect response to the different drugs based on their mechanism of action or other biochemical properties. GWAS results are publicly available for all SNPs tested at the Plenge laboratory and RICOPILI Web sites (see URLs). GWAS results for all SNPs achieving P<10^−6^ from any analysis are detailed in the Table S3.

**Figure 2:**
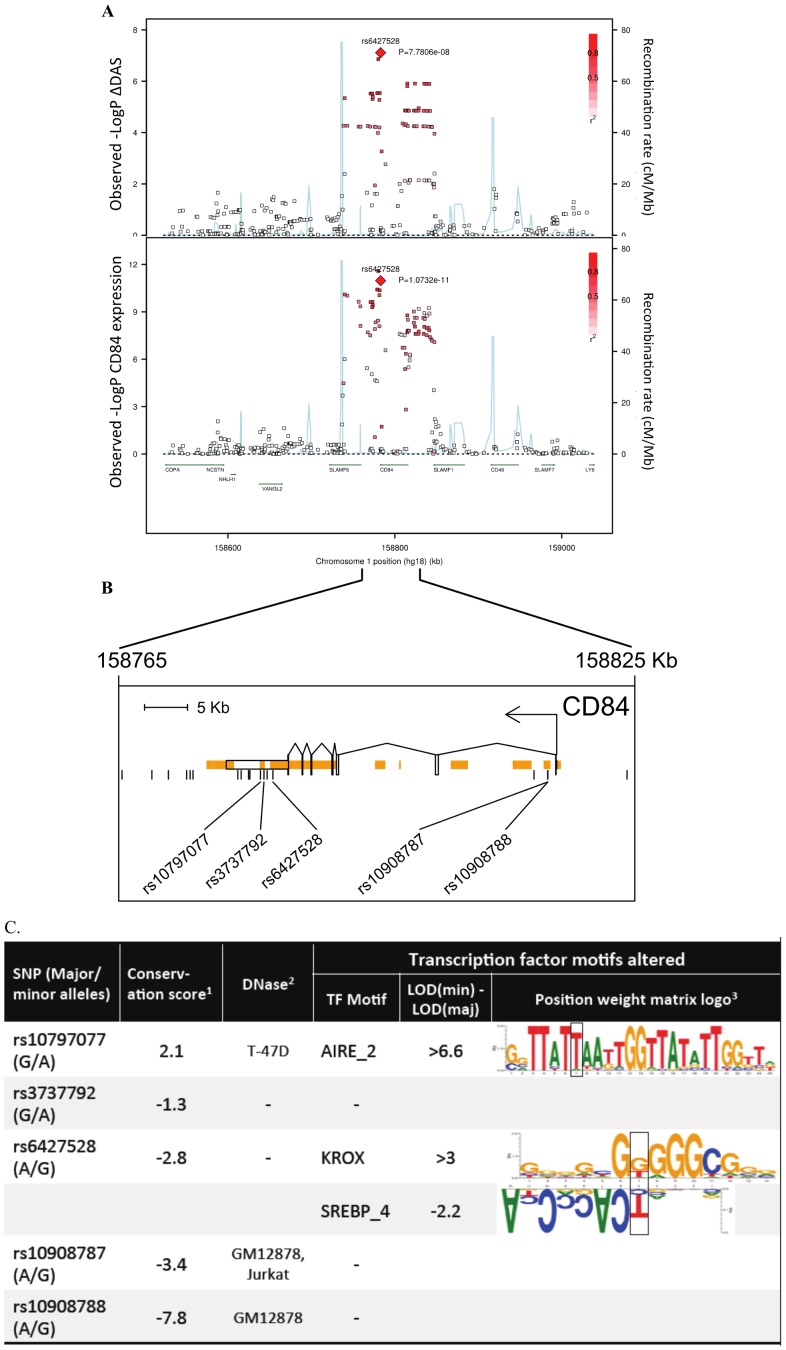
Association results and SNP annotations in the *1q23 CD84* locus. A) Regional association plots with ΔDAS (top panel) and with *CD84* expression (bottom panel), showing strengths of association (−Log10 P-value) versus position (Kb) along chromosome 1. B) Schematic of *CD84* gene structure (RefSeq gene model, box exons connected by diagonal lines, arrow indicates direction of transcription) with strong enhancer chromatin states (orange rectangles) and SNPs in high LD (r2>0.8) with rs6427528 (vertical ticks). SNPs in enhancers are labeled below. C) Annotations of strong-enhancer rs6427528 proxy SNPs; listed are SNP rs-ID (major and minor alleles), conservation score, cell line with DNAse footprint if present, and transcription factor binding sites altered. 1- Genomic evolutionary rate profiling (GERP) conservation score, where a score >2 indicates conservation across mammals. 2- DNase footprint data are compiled from publicly available experiments by HaploReg. 3- Position weight matrix logos show transcription factor consensus binding sites with nucleotide bases proportional to binding importance. SNP position is boxed. Note that the rs10797077 AIRE_2 and the rs6427528 SREBP_4 motifs are on the minus strand (base complements correspond to SNP alleles), with the SREBP motif shown upside down to align with the rs6427528 KROX motif on the positive strand. Data are from HaploReg.

**Figure 3:**
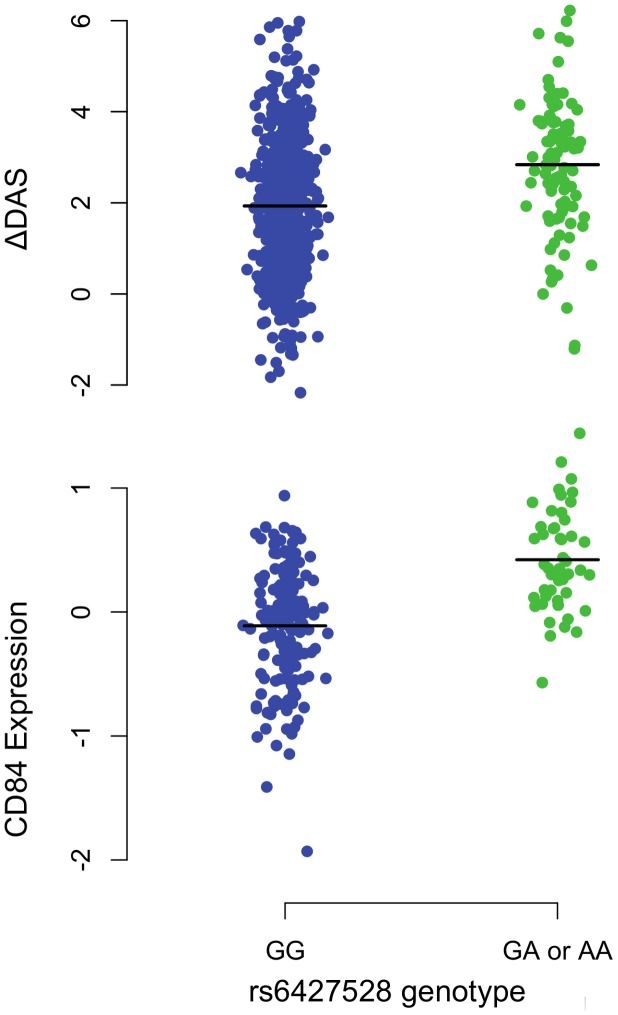
*1q23/CD84* genotype association plots for ΔDAS and *CD84* gene expression. Shown are ΔDAS in our GWAS in etanercept-treated patients (top panel, n = 733; n = 634 with the GG genotype and n = 99 with the GA or AA genotype) and *CD84* expression in our eQTL results (bottom panel, n = 228 non-RA patients; n = 178 with the GG genotype and n = 50 with the GA or AA genotype). The rare-allele homozygous genotype AA was observed four times in our ΔDAS GWAS and was pooled with the heterozygous GA genotype for this figure; AA homozygotes were not observed in the *CD84* eQTL data. Association analyses reported in the text regressed phenotype (ΔDAS, P = 8×10^−8^; *CD84* expression, P = 1×10^−11^) on minor-allele dosage (range 0–2).

For etanercept-treated RA patients, a locus on chromosome *1q23* achieved near-genome-wide significance (rs6427528, P_META_ = 8×10^−8^) (Figure 1B, Figure 2A, and Figure 3), but not in the infliximab or adalimumab subsets (*P*>0.05) (Figure S3). SNPs in linkage disequilibrium (LD) showed consistent association results (rs1503860, P = 1×10^−7^, r^2^ = 1 with rs6427528 in HapMap; three perfect-LD clusters of SNPs exemplified by rs3737792, rs10908787 and rs11265432 respectively; P<5×10^−6^; r^2^ = 0.83, 0.63 and 0.59 with rs6427528, respectively). No single collection was responsible for the signal of association, as the effect size was consistent across all collections (Figure S4). The top SNP rs6427528 was genotyped in the ReAct dataset (Illumina Omni Express genotyping chip), and was well imputed across all other datasets (imputation quality score INFO ≥0.94, which is an estimate of genotype accuracy; the range of INFO scores is 0–1, where 1 indicates high confidence). All of these SNPs had minor allele frequencies ranging from 7–10%. The SNP explains 2.6% variance in response to etanercept treatment.

For patients treated with infliximab, we observed a suggestive result on chromosome *10p14* (rs12570744, P = 2×10^−7^). No highly significant or suggestive results were observed for the ΔDAS phenotype in patients treated with adalimumab (P_META_>10^−5^).

Qualitatively similar results were attained in the analysis of our secondary phenotype, EULAR good responder vs non-responder status (Figures S1 and S2). For SNPs at the *1q23* locus, the pattern of association with responder/non-responder status (etanercept-treated patients) was consistent with the results for ΔDAS (P = 6×10^−3^ for rs6427528 and rs1503860). We also identified potential novel associations, with suggestive results for infliximab (rs4336372, chromosome *5q35*, P = 8×10^−7^) and adalimumab (rs940928, chromosome *2q12*, P = 2×10^−6^).

### eQTL and sequence analysis of the *CD84* gene

For each SNP with P<10^−6^ identified by our GWAS (n = 6 independent SNPs), we searched for biological evidence to support a true positive association. We used genome-wide sequence data from the 1000 Genomes Project to search for putative functional variants in LD with the index SNP (defined as SNPs predicted to change protein-sequence or mRNA splicing). We also used genome-wide expression data to search for an expression quantitative trait locus (eQTL) in public databases and in peripheral blood mononuclear cells (PBMCs) in 228 non-RA patients and in 132 RA patients.

While we did not identify any variants disrupting protein-coding sequences or mRNA splicing, we did find that the *1q23* SNP associated with response to etanercept therapy was a strong eQTL in PBMCs (Figure 2A and Figure 3). In an analysis of 679 SNPs for cis-regulated expression of five genes in the region of LD (*SLAMF6*, *CD84*, *SLAMF1*, *CD48*, and *SLAMF7*), we found that rs6427528-*CD84* (and SNPs in LD with it) was the top eQTL of all results (n = 228 subjects; Figure 2A). This SNP was specifically associated with *CD84* expression, and was not an eQTL for other genes in the region (P>0.36 for the other genes).

We replicated our eQTL finding in 132 RA patients with both GWAS data and genome-wide expression data. PBMC expression data were available from RA patients in the Brigham RA Sequential Study (BRASS) and Autoimmune Biomarkers Collaborative Network (ABCoN) collections. We observed a significant association between rs6427528 genotype and *CD84* expression (linear regression adjusted for cohort P = 0.004, rank correlation P = 0.018). The direction of effect was the same as in the PBMC samples from 228 non-RA patients. A combined analysis of RA patients and the non-RA patient eQTL data (described above) yielded rank correlation P = 3×10^−10^ (n = 360 total individuals).

We searched sequence data to determine if rs6427528, or any of the SNPs in LD with it, were located within conserved, non-coding motifs that might explain the eQTL data. We used HaploReg [16] to examine the chromatin context of rs6427528 and 26 SNPs in LD with it (at r^2^>0.50). We found that 5 SNPs occur in strong enhancers inferred from chromatin marks (Figure 2B) [17]. Two of these 5 SNPs, rs10797077 and rs6427528 (r^2^ = 0.74 to each other), are predicted to disrupt transcription factor binding sites, and rs10797077 occurs at a site that shows conservation across mammalian genomes [18]. Figure 2C shows the DNA sequence position weight matrices of the transcription factor binding sites changed by rs10797077 (the minor allele creates a stronger binding site for the AIRE transcription factor) and rs6427528 (the minor allele creates a binding site for KROX and SREBP).

### Expression of *CD84* as a biomarker of disease activity and treatment response

**Figure 4:**
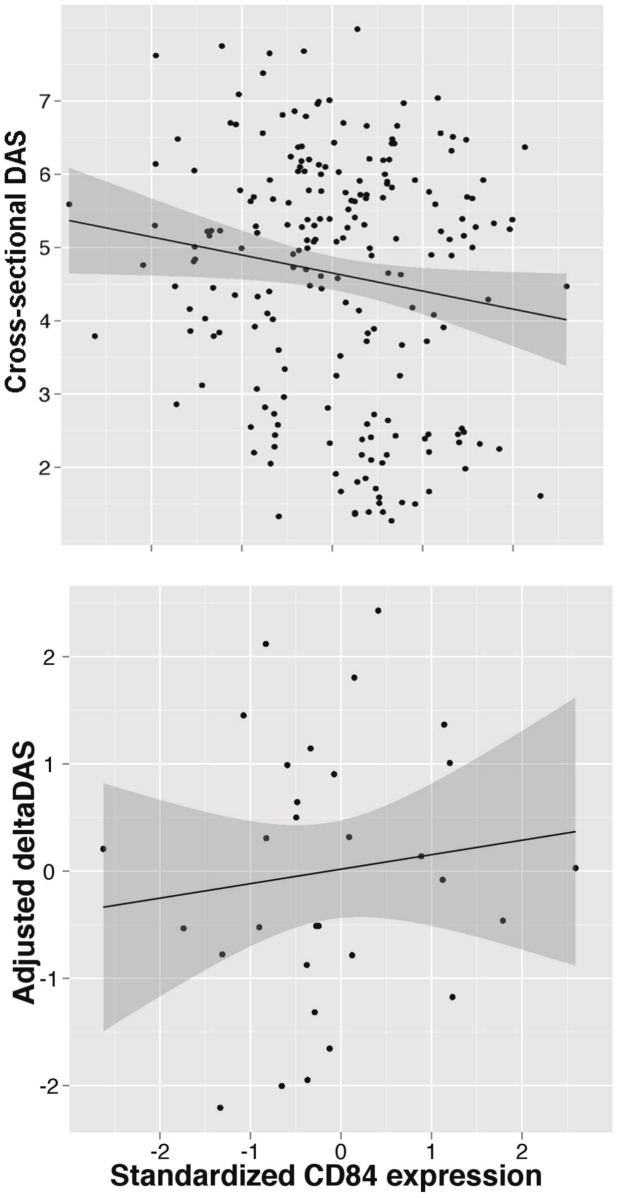
*CD84* expression level and clinical features. Analyses are shown in RA patients from the BRASS and ABCoN registries, for baseline DAS (top panel, n = 210; R^2^ = 0.02, p = 0.02) and ΔDAS (bottom panel, n = 31; R^2^ = 0.001, p = 0.46). Best-fit linear regression lines are shown in black, with shaded regions showing linear regression model (slope and intercept) 95% confidence intervals. *CD84* expression levels were quantile normalized, and ΔDAS values were adjusted for age, gender and baseline DAS.

Because the genetic data demonstrates that the allele associated with better response is associated with higher *CD84* expression, this suggests that *CD84* expression itself may serve as a useful biomarker of disease activity or treatment response. We tested both hypotheses using PBMC expression data from the BRASS and ABCoN collections. First, we tested if *CD84* expression is associated with cross-sectional DAS, adjusting for age, gender and cohort (Figure 4). We observed a significant inverse association between *CD84* expression and cross-sectional DAS in 210 RA patients (beta = −0.3, P = 0.02, r^2^ = 0.02). That is, higher *CD84* expression was associated with lower DAS, regardless of treatment.

Second, we tested *CD84* for association with our primary treatment response phenotype, ΔDAS. The sample size for this analysis was smaller than for the cross-sectional analysis, as we required that patients be on anti-TNF therapy and have pre- and post-treatment DAS. We found that *CD84* expression levels showed a non-significant trend towards an association with ΔDAS in 31 etanercept-treated patients (beta = 0.2, r^2^ = 0.002, P = 0.46) and in all 78 anti-TNF-treated patients (beta = 0.14, r^2^ = 0.004, P = 0.4). The effect is in the same direction one would predict based on the genetic association at rs6427528: the allele associated with better response is also associated with higher *CD84* expression (Figure 3), and in 31 RA patients, higher *CD84* expression (regardless of genotype) is associated with a larger ΔDAS (i.e., better response; Figure 4).

### Replication of genetic data in a small, multi-ethnic cohort

**Figure 5:**
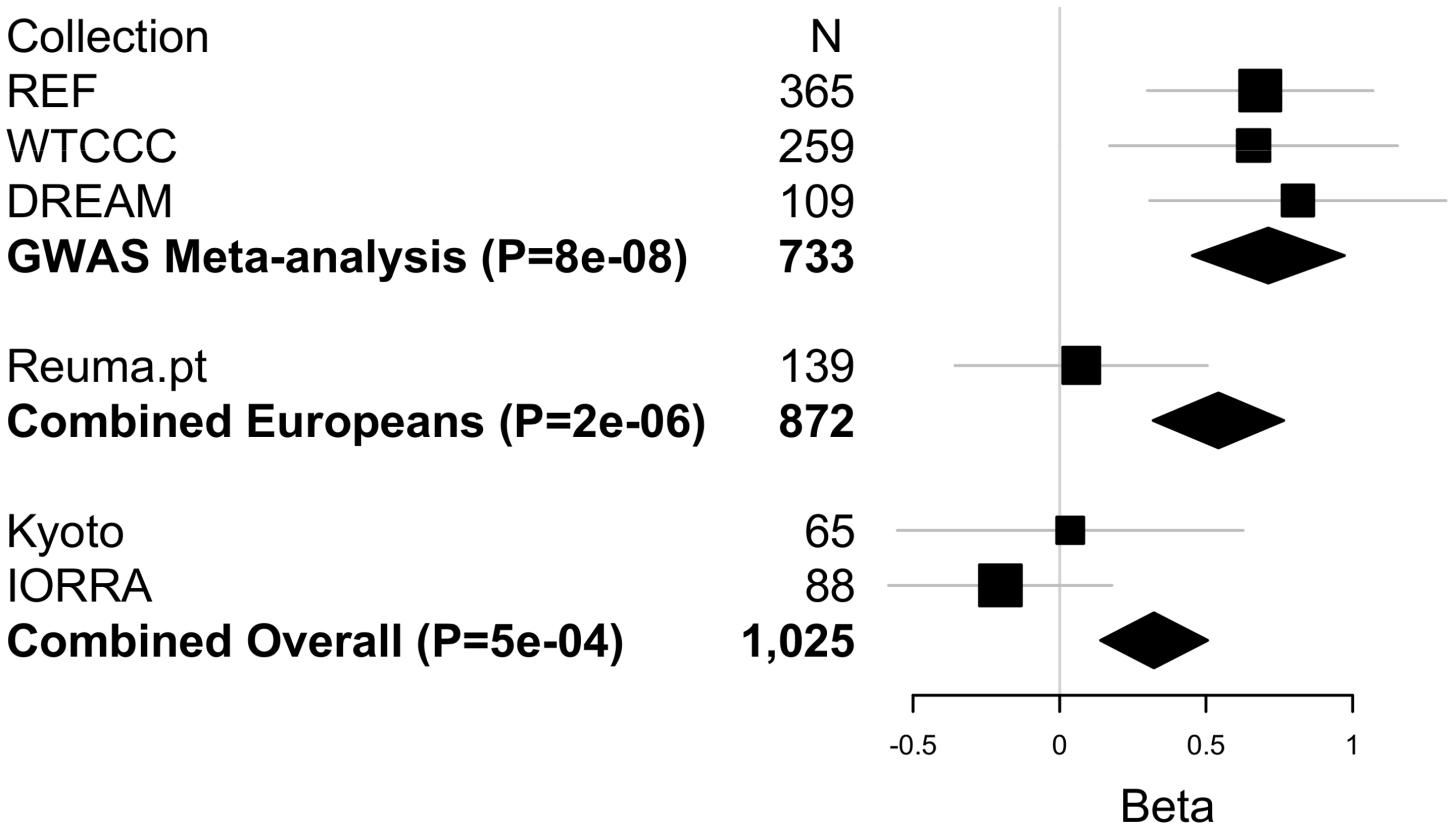
Replication and overall results for the *CD84* SNP rs6427528. Forest plot shows each cohort, sample size and linear regression beta coefficient estimates with symbol size proportional to cohort sample size and thin horizontal lines showing beta 95% CIs. Inverse variance weighted meta-analysis results are shown in bold for GWAS, GWAS+European (Portuguese) replication samples, and for GWAS+European+Asian (Japanese) replication samples, with diamond widths indicating beta 95% CIs.

Since most of the samples available to us as part of our international collaboration were included in our GWAS, few additional samples were available for replication. In addition, the remaining samples available to us were from different ethnic backgrounds. Nonetheless, we sought to replicate the associations of rs6427528 with ΔDAS in these additional samples. We genotyped 139 etanercept-treated patients from a rheumatoid arthritis registry in Portugal (Reuma.pt) and 151 etanercept-treated patients from two Japanese collections (IORRA, n = 88 patients on etanercept and Kyoto University, n = 63 on etanercept). Replication sample sizes, clinical data and results for these two SNPs are shown in Table S4. Based on the observed effect size in the GWAS and observed allele frequency in the replication samples, we had 32% power to replicate this finding in the Portuguese samples and 17% power to replicate this finding in the Asian samples at P<0.05. The same association analysis as for GWAS was carried out: linear regression assuming an additive genetic model and using ΔDAS as phenotype, adjusted for baseline DAS. Replication results are shown in Figure 5.

While the SNPs fail to replicate in these patient collections at P<0.05, the direction of effect is the same in the Portuguese and Kyoto replication samples as in our GWAS. In a combined analysis limited to subjects of European-ancestry (GWAS data and Portuguese replication samples), rs6427528 remained highly suggestive (P = 2×10^−6^). Including the Japanese subjects, the overall GWAS+replication combined meta-analysis P-value remained suggestive (P = 5×10^−4^).

## Discussion

Here we present the largest GWAS to date on anti-TNF therapy response in 2,706 RA patients. We find a significant association at the *1q23*/*CD84* locus in 733 etanercept treated patients (P = 8×10^−8^), but not in RA patients treated with drugs that act as a monoclonal antibody to neutralize TNF (infliximab or adalimumab). The allele associated with a larger ΔDAS (i.e., better response) was associated with higher *CD84* expression in PBMCs from non-RA patients (P = 1×10^−11^) and in RA patients (P = 0.004).

We first conducted a GWAS of both categories of anti-TNF drugs (the soluble receptor drug, etanercept, and two monoclonal antibody drugs, infliximab and adalimumab). However, this analysis revealed no strongly associated SNPs. When we subset our GWAS by each of the three individual drugs, several SNPs in the *1q23* locus were highly significant in etanercept-treated patients, and SNPs in three other loci (*10p15*, *5q35* and *2q12*) were associated in infliximab or adalimumab subset analyses. Furthermore, the top SNPs for each analysis (Table S3) showed little correlation across the three anti-TNF drugs. This simple observation suggests that genetic control of treatment response may be different for different drugs. This finding is consistent with the clinical observation that RA patients who fail one anti-TNF drug may still respond to a different anti-TNF drug, albeit at lower rates of response [19]. If confirmed in larger samples and more comprehensive analyses, then this could have major implications for how physicians prescribe these drugs.

The most significant finding from our GWAS was a set of equivalent SNPs in LD with each other from the *1q23* locus in etanercept-treated RA patients (Figure 1 and Figure 2A). While the top SNP did not reach genome-wide significance in predicting treatment response, it did reach genome-wide significance as an eQTL in PBMCs (P = 1×10^−11^; Figure 2A). This finding indicates that the SNP (or another variant in LD with it) is indeed biologically functional in a human tissue that is important in the immune response. Two SNPs, rs10797077 and rs6427528, disrupt transcription factor binding sites, and represent excellent candidates for the causative allele to explain the effect on *CD84* expression (Figure 2C).

Our findings suggest that *CD84* genotype and/or expression could be a biomarker for etanercept treatment response among individuals of European ancestry. The genetic and expression data predict that *CD84* expression should be positively associated with treatment response (i.e., higher expression is associated with better response; Figure 3). While we did not observe a significant association between *CD84* levels and ΔDAS, we did observe a trend consistent with this prediction (Figure 4). Importantly, we note that power was extremely limited with the small sample sizes for which we had *CD84* expression as well as drug response data (n = 31 RA patients treated with etanercept).

The *CD84* gene is a compelling candidate for immune response, belonging to the CD2 subset of the immunoglobulin superfamily. It has been implicated in T-cell activation and maturation [20]. CD84 localizes to the surface of CD4+ and CD8+ T cells, and acts as a costimulatory molecule for IFN-gamma secretion [21]. *CD84* is also expressed in B-cells, monocytes and platelets. *CD84* has not been previously implicated in genetic studies of RA risk, disease activity, disease severity, or treatment response.

A limitation of our study is the small sample size available for replication (n = 290 etanercept-treated patients), and the lack of replication observed for the top *CD84* SNP (rs6427528) among patients of Portuguese and Japanese ancestry. The simplest explanation is that our original observation in the GWAS data represents a false positive association. However, the eQTL and gene expression data argue against this possibility. Explanations for a false negative finding in our replication collections include: (1) lack of power, especially if the effect size observed in the GWAS represents an over-estimate of the true effect size (the Winner's Curse) – we estimate that we had 32% and 17% power (at *P* = 0.05) to detect an association in the Portuguese and Japanese sample collections, respectively; (2) clinical heterogeneity, which is always a possibility in pharmacogenetic studies, especially those conducted in different countries; and (3) ethnic differences, including different patterns of LD between the underlying causative allele (which is as yet unknown) and marker SNPs tested in our study. We did observe subtle differences in local patterns of LD between Asians and Europeans using genetic data from the 1000 Genomes Project (Figure S5). We note that the rs6427528 minor allele A has a frequency of ∼5–10% in European and East Asian populations, and ∼50% in the African YRI population (HapMap2 and 1000 Genomes); therefore, it may be of interest to test African American samples in replication.

What are the options for increasing sample size in pharmacogenetic studies, thereby providing an opportunity to replicate our *CD84* genetic and expression findings? While it might seem trivial to collect more samples through traditional registries, this is extremely challenging for phenotypes pertaining to treatment efficacy. To underscore this point, we highlight our study design, where we organized samples and clinical data from 16 different collections across 7 different countries in order to obtain the samples for the current study. Going forward, non-traditional strategies to collect biospecimens linked with clinical data (e.g., online registries, electronic medical records) may be required to achieve clinical collections of sufficient size to discover pharmacogenomic predictors of efficacy.

In conclusion, we conducted the largest GWAS to date for response to anti-TNF therapy in RA patients. Our genetic and expression data suggest that *CD84* genetic variants and/or expression levels could be developed as predictive biomarkers for etanercept treatment response in RA patients of European ancestry.

## Methods

### Samples and clinical data

All patients met 1987 ACR criteria for RA, or were diagnosed by a board-certified rheumatologist. In addition, patients were required to have at least moderate disease activity at baseline (DAS>3.2). All patients gave their informed consent and all institutional review boards approved of this study. A total of 13 collections from across 5 countries were included in GWAS [11], [12], [13], [22]: Autoimmune Biomarkers Collaborative Network (ABCoN) from the U.S. (N = 79); the Genetics Network Rheumatology Amsterdam (GENRA, N = 53); the Dutch Behandelstrategieen voor Rheumatoide Arthritis (BeSt, N = 85); the U.K. Biological in Rheumatoid arthritis Genetics and Genomics Study Syndicate (BRAGGSS, N = 140); the U.S. Brigham Rheumatoid Arthritis Sequential Study (BRASS, N = 55); the Swedish Epidemiological Investigation of Rheumatoid Arthritis (EIRA, N = 298); the Immunex Early Rheumatoid Arthritis study (eRA N = 57); the Swedish Karolinska Institutet study (KI, N = 77); the Netherlands collection from Leiden University Medical Center (LUMC, N = 43); and the U.S. Treatment of Early Aggressive RA (TEAR, N = 109). We refer to these collections as the American College of Rheumatology Research and Education Foundation (REF) collection, as funding for GWAS genotyping was provided by the “*Within Our Reach*” project. We included additional samples from BRAGGSS (N = 595) [12]; the Dutch Rheumatoid Arthritis Monitoring registry (DREAM) in the Netherlands, and the ApotheekZorg (AZ) database (which facilitates the Dutch distribution of adalimumab; N = 880) [23], [24], together referred to as DREAM; and the French Research in Active Rheumatoid Arthritis (ReAct, N = 272) [25].

Additional samples were collected for replication of SNPs in the *1q23* locus. These included the Rheumatic Diseases Portuguese Register (Reuma.pt, N = 378) from the Portuguese Society of Rheumatology (SPR), which captures more than 90% of patients treated with biological therapies and managed in rheumatology departments across Portugal [26]. Additional replication samples (N = 374) of East Asian ancestry were included from the IORRA and Kyoto University Hospital registries, part of the Japanese Genetics and Allied research in Rheumatic diseases Networking consortium (GARNET) [27].

Clinical data were collected in each cohort, including disease activity scores at baseline and at least one time point after treatment, gender, age, methotrexate use, as well as autoantibody status (RF or CCP). The composite disease activity scores for 28 joints (DAS28) included laboratory values for erythrocyte sedimentation rate (ESR) for most samples and C-reactive protein (CRP) for 191 samples in the REF collection (ABCoN, BRASS and eRA cohorts). DAS28 values were available at baseline and at 3–12 months after initiating anti-TNF therapy. Our primary phenotype was defined as ΔDAS = baseline DAS - end DAS, and responder status was also determined according to EULAR criteria for start and end DAS [15]. Clinical variables were assessed for association with phenotype in multivariate linear or logistic regression models for both the ΔDAS and EULAR responder-status phenotypes. Clinical variables that were significant in these analyses were retained as covariates in genetic association tests, except for methotrexate co-therapy. Including a covariate for methotrexate co-therapy reduced sample size substantially due to missing clinical data, so results were compared for our primary analysis and a secondary analysis with the covariates (and with reduced sample size) and the results were verified not to be impacted (not shown).

### Genotyping and data processing

A total of eleven genotyping batches were processed separately. (1) BRASS samples were genotyped using Affymetrix 6.0 chip [28]; (2) WTCCC samples were genotyped on Affymetrix 500K chip [12]. All other cohorts were genotyped using Illumina platform arrays (see Table 1). Our American College of Rheumatology Research Education Fund (REF) collection was made up of smaller cohorts from throughout North America and Europe, including BRASS samples. Also included in REF: (3) ABCoN [13] and (4) EIRA [29] were separately genotyped on the Illumina 317K genotyping array; (5) eRA on the Illumina 550K chip; and (6) GENRA, BeSt, BRAGGSS (a subset of N = 53 samples), KI and LUMC were genotyped in one batch, and (7) BRAGGSS (N = 87) and TEAR were genotyped in a second batch, both using Illumina 660k chips, at the Broad Institute (8–10). DREAM and AZ samples were genotyped in three batches, one on 550K chip and two on 660K chips (manuscript in preparation), and (11) ReAct samples were genotyped on Illumina OmniExpress chips. Quality control (QC) filtering was done in each genotyping batch, including filtering individuals with >5% missing data, and filtering SNPs with >1% missing data, minor allele frequency (MAF) <1% and Chi-squared test of Hardy Weinberg equilibrium *P_HWE_*<10^−5^. We then used individual-pairwise identity-by-state estimates to remove occasional related and potentially contaminated samples. Data processing and QC were performed in PLINK [30]. Principal Components Analysis (PCA) was performed using EIGENSTRAT [31] (default settings) on the combined dataset using 20,411 SNPs genotyped across all datasets. Ethnicity outliers including all individuals of non-European decent were identified and removed, and the first three eigenvectors were used as covariates in GWAS.

Imputation was conducted on each of eleven datasets separately, using the IMPUTE v1 software [32] and haplotype-phased HapMap Phase 2 (release 22) European CEU founders as a reference panel. Imputation of BRASS and EIRA was previously reported [28], [33], and we followed the same imputation procedures for the remaining datasets. Imputation yielded posterior genotype probabilities as well as imputation quality scores at SNPs not genotyped with a minor allele frequency ≥1% in HapMap CEU. We removed imputed SNPs with imputation ‘info’ scores <0.5 or MAF <1% in any of the datasets.

### Expression profile and eQTL data

Gene expression levels were quantified using mRNA derived from peripheral blood mononuclear cells (PBMCs) using Affymetrix Human Genome U133 Plus 2.0, for 255 multiple sclerosis patients in the Comprehensive Longitudinal Investigation of MS at the Brigham and Women's Hospital [34], either untreated (N = 83) or treated with interferon-beta (N = 105) or glatiramer acetate (N = 67). The raw intensity values were subject to quality control based on the recommended pipeline available in the simpleaffy and affyPLM R Bioconductor packages, and were then normalized using GCRMA (N = 228). The data are available on the Gene Expression Omnibus website (GSE16214). Expression levels for 17,390 probes mapping to 9,665 Ensembl transcripts were adjusted for confounding factors including age, gender, drug and batch using principle components and Bayesian factor analysis [35], and used in eQTL association analyses. Genotype data were collected on the Affymetrix 550K GeneChip 6.0 platform as a part of a previously published study [36]. Allelic dosages from imputed data (HapMap Phase II CEU samples; >2 million SNPs, MACH imputation quality >0.1 and MAF> = 0.05) were used for association analysis. *Cis*-eQTLs were identified +/−1 Mb of transcription start sites (TSS) in the *1q23* locus region. Significance was evaluated by 10,000 permutations per gene, and false discovery rates were calculated based on cis-eQTL analyses in the total of 9,665 genes [37].

Additional expression profile data were available for subsets of samples that were part of two cohorts in our GWAS. Expression data from patients enrolled in the BRASS registry have been previously published [38]. Expression data were collected on Affymetrix Gene Chip U133 Plus 2 microarrays. BRASS patients had either cross-sectional expression data (n = 132, assayed at the time the patient was enrolled in BRASS) or pre- and post-treatment expression data (n = 17 samples, 8 treated with etanercept). Of these, n = 87 patients had expression and GWAS data. For patients with pre- and post-treatment data, we used the “baseline” pre-treatment expression data for cross-sectional analysis. In ABCoN, 65 RA patients (n = 23 treated with etanercept) had both pre- and post-treatment expression data, as well as ΔDAS clinical data [39], and n = 45 patients had expression and GWAS data. As with BRASS, we use the “baseline” pre-treatment expression data for cross-sectional analysis. For ABCoN expression profile data were collected on Illumina Human WG6v3 microarrays and were quantile normalized according to Illumina recommended protocols. Within both BRASS and ABCoN, expression data were normalized to the mean and standard deviation within each collection. For prospective analyses of expression data and ΔDAS, we combined BRASS and ABCoN to include 31 etanercept-treated patients and 78 anti-TNF-treated patients.

### Statistical analyses

In our primary GWAS analysis, we tested each SNP for association with ΔDAS using linear regression adjusted for baseline DAS and the first 3 PCA eigenvectors in each collection. In our secondary GWAS analysis, we modeled SNPs predicting EULAR good response *versus* EULAR non-response using logistic regression, again adjusting for start-DAS value and the first three eigenvectors. Association analysis was done using SNPTEST [32] assuming an additive genetic model. Genomic control *λ*_GC_ values [40] for genotyped SNPs only and all SNPs were calculated, and no inflation or deflation was observed in the distributions of association test results. We then conducted inverse variance-weighted meta-analysis to combine results across the four datasets, and conducted Cochran's Q tests for heterogeneity using the β coefficients [41]. We further divided samples into 3 subsets according to drug (etanercept, infliximab or adalimumab). GWAS analysis for each group followed the same analysis procedure. Meta-analysis and heterogeneity tests were conducted using SAS. Expression analyses utilized linear regression or Spearman rank correlation, also using SAS. We tested for effects of cohort, age, gender and concurrent methotrexate, and results are shown using significant covariates as indicated.

## Supporting Information

Figure S1 Quantile–quantile (QQ) plots for ΔDAS and response analysis, with genomic control λ_GC_ values.
(TIF)

Figure S2 GWAS results for the good response versus non-response phenotype. Shown are strengths of association (−Log10 P-value) for each SNP versus position along chromosomes 1 to 22. A) All samples (n = 1,708). B) Etanercept-treated patients (n = 472). C) Infliximab-treated patients (n = 599). D) Adalimumab-treated patients (n = 636).
(TIF)

Figure S3 Forest plot of replication results for the CD84 SNP rs6427528, in patients treated with anti-TNF drugs other than etanercept (infliximab & adalimumab).
(TIF)

Figure S4 Forest plot of CD84 result in patients treated with etanercept, subset by all collections.
(TIF)

Figure S5 Patterns of linkage disequilibrium (LD) at the CD84 locus in HapMap. Shown patterns of LD for CEU (top panel) and CHBJPT (bottom panel).
(TIF)

Table S1 Sample information for each of thirteen clinical batches.
(DOC)

Table S2 Clinical multivariate model for the ΔDAS phenotype.
(DOC)

Table S3 GWAS results for all SNPs achieving P<10^−6^ from any analysis.
(XLS)

Table S4 Sample and clinical data summary for replication samples.
(DOC)

## References

[1] Klareskog L, Catrina AI, Paget S (2009) Rheumatoid arthritis. Lancet 373: 659–672. 19157532 10.1016/S0140-6736(09)60008-8

[2] Scott DL, Wolfe F, Huizinga TW (2010) Rheumatoid arthritis. Lancet 376: 1094–1108. 20870100 10.1016/S0140-6736(10)60826-4

[3] McInnes IB, Schett G (2011) The pathogenesis of rheumatoid arthritis. N Engl J Med 365: 2205–2219. 22150039 10.1056/NEJMra1004965

[4] Aeberli D, Seitz M, Juni P, Villiger PM (2005) Increase of peripheral CXCR3 positive T lymphocytes upon treatment of RA patients with TNF-alpha inhibitors. Rheumatology (Oxford) 44: 172–175. 15509629 10.1093/rheumatology/keh437

[5] Agnholt J, Dahlerup JF, Kaltoft K (2003) The effect of etanercept and infliximab on the production of tumour necrosis factor alpha, interferon-gamma and GM-CSF in in vivo activated intestinal T lymphocyte cultures. Cytokine 23: 76–85. 12906870 10.1016/s1043-4666(03)00201-1

[6] Catrina AI, Trollmo C, af Klint E, Engstrom M, Lampa J, et al (2005) Evidence that anti-tumor necrosis factor therapy with both etanercept and infliximab induces apoptosis in macrophages, but not lymphocytes, in rheumatoid arthritis joints: extended report. Arthritis Rheum 52: 61–72. 15641091 10.1002/art.20764

[7] Scallon BJ, Moore MA, Trinh H, Knight DM, Ghrayeb J (1995) Chimeric anti-TNF-alpha monoclonal antibody cA2 binds recombinant transmembrane TNF-alpha and activates immune effector functions. Cytokine 7: 251–259. 7640345 10.1006/cyto.1995.0029

[8] Gudbrandsdottir S, Larsen R, Sorensen LK, Nielsen S, Hansen MB, et al (2004) TNF and LT binding capacities in the plasma of arthritis patients: effect of etanercept treatment in juvenile idiopathic arthritis. Clin Exp Rheumatol 22: 118–124. 15005015

[9] Plant D, Prajapati R, Hyrich KL, Morgan AW, Wilson AG, et al (2012) Replication of association of the PTPRC gene with response to anti-tumor necrosis factor therapy in a large UK cohort. Arthritis Rheum 64: 665–670. 21952740 10.1002/art.33381 PMC3427899

[10] Prajapati R, Plant D, Barton A (2011) Genetic and genomic predictors of anti-TNF response. Pharmacogenomics 12: 1571–1585. 22044414 10.2217/pgs.11.114

[11] Cui J, Saevarsdottir S, Thomson B, Padyukov L, van der Helm-Van Mil AH, et al (2010) Rheumatoid arthritis risk allele PTPRC is also associated with response to anti-tumor necrosis factor alpha therapy. Arthritis Rheum 62: 1849–1861. 20309874 10.1002/art.27457 PMC3652476

[12] Plant D, Bowes J, Potter C, Hyrich KL, Morgan AW, et al (2011) Genome-wide association study of genetic predictors of anti-tumor necrosis factor treatment efficacy in rheumatoid arthritis identifies associations with polymorphisms at seven loci. Arthritis Rheum 63: 645–653. 21061259 10.1002/art.30130 PMC3084508

[13] Liu C, Batliwalla F, Li W, Lee A, Roubenoff R, et al (2008) Genome-wide association scan identifies candidate polymorphisms associated with differential response to anti-TNF treatment in rheumatoid arthritis. Mol Med 14: 575–581. 18615156 10.2119/2008-00056.Liu PMC2276142

[14] Prevoo ML, van 't Hof MA, Kuper HH, van Leeuwen MA, van de Putte LB, et al (1995) Modified disease activity scores that include twenty-eight-joint counts. Development and validation in a prospective longitudinal study of patients with rheumatoid arthritis. Arthritis Rheum 38: 44–48. 7818570 10.1002/art.1780380107

[15] van Gestel AM, Prevoo ML, van 't Hof MA, van Rijswijk MH, van de Putte LB, et al (1996) Development and validation of the European League Against Rheumatism response criteria for rheumatoid arthritis. Comparison with the preliminary American College of Rheumatology and the World Health Organization/International League Against Rheumatism Criteria. Arthritis Rheum 39: 34–40. 8546736 10.1002/art.1780390105

[16] Ward LD, Kellis M (2012) HaploReg: a resource for exploring chromatin states, conservation, and regulatory motif alterations within sets of genetically linked variants. Nucleic Acids Res 40: D930–934. 22064851 10.1093/nar/gkr917 PMC3245002

[17] Ernst J, Kheradpour P, Mikkelsen TS, Shoresh N, Ward LD, et al (2011) Mapping and analysis of chromatin state dynamics in nine human cell types. Nature 473: 43–49. 21441907 10.1038/nature09906 PMC3088773

[18] Cooper GM, Stone EA, Asimenos G, Green ED, Batzoglou S, et al (2005) Distribution and intensity of constraint in mammalian genomic sequence. Genome Res 15: 901–913. 15965027 10.1101/gr.3577405 PMC1172034

[19] Soliman MM, Hyrich KL, Lunt M, Watson KD, Symmons DP, et al (2012) Rituximab or a second anti-TNF therapy for rheumatoid arthritis patients who have failed their first anti-TNF? Comparative analysis from the British Society for Rheumatology Biologics Register. Arthritis Care Res (Hoboken) 10.1002/acr.21663 PMC3492906 22422731

[20] Tangye SG, Nichols KE, Hare NJ, van de Weerdt BC (2003) Functional requirements for interactions between CD84 and Src homology 2 domain-containing proteins and their contribution to human T cell activation. J Immunol 171: 2485–2495. 12928397 10.4049/jimmunol.171.5.2485

[21] Martin M, Romero X, de la Fuente MA, Tovar V, Zapater N, et al (2001) CD84 functions as a homophilic adhesion molecule and enhances IFN-gamma secretion: adhesion is mediated by Ig-like domain 1. J Immunol 167: 3668–3676. 11564780 10.4049/jimmunol.167.7.3668

[22] Padyukov L, Lampa J, Heimburger M, Ernestam S, Cederholm T, et al (2003) Genetic markers for the efficacy of tumour necrosis factor blocking therapy in rheumatoid arthritis. Ann Rheum Dis 62: 526–529. 12759288 10.1136/ard.62.6.526 PMC1754569

[23] Coenen MJ, Enevold C, Barrera P, Schijvenaars MM, Toonen EJ, et al (2010) Genetic variants in toll-like receptors are not associated with rheumatoid arthritis susceptibility or anti-tumour necrosis factor treatment outcome. PLoS ONE 5: e14326 doi:10.1371/journal.pone.0014326. 21179534 10.1371/journal.pone.0014326 PMC3002281

[24] Toonen EJ, Coenen MJ, Kievit W, Fransen J, Eijsbouts AM, et al (2008) The tumour necrosis factor receptor superfamily member 1b 676T>G polymorphism in relation to response to infliximab and adalimumab treatment and disease severity in rheumatoid arthritis. Ann Rheum Dis 67: 1174–1177. 18385279 10.1136/ard.2008.088138

[25] Miceli-Richard C, Comets E, Verstuyft C, Tamouza R, Loiseau P, et al (2008) A single tumour necrosis factor haplotype influences the response to adalimumab in rheumatoid arthritis. Ann Rheum Dis 67: 478–484. 17673491 10.1136/ard.2007.074104 PMC2750008

[26] Canhao H, Faustino A, Martins F, Fonseca JE (2011) Reuma.pt - the rheumatic diseases portuguese register. Acta Reumatol Port 36: 45–56. 21483280

[27] Okada Y, Terao C, Ikari K, Kochi Y, Ohmura K, et al (2012) Meta-analysis identifies nine new loci associated with rheumatoid arthritis in the Japanese population. Nat Genet 10.1038/ng.2231 22446963

[28] Stahl EA, Raychaudhuri S, Remmers EF, Xie G, Eyre S, et al (2010) Genome-wide association study meta-analysis identifies seven new rheumatoid arthritis risk loci. Nat Genet 42: 508–514. 20453842 10.1038/ng.582 PMC4243840

[29] Plenge RM, Seielstad M, Padyukov L, Lee AT, Remmers EF, et al (2007) TRAF1-C5 as a Risk Locus for Rheumatoid Arthritis – A Genomewide Study. N Engl J Med 357: 1199–1209. 17804836 10.1056/NEJMoa073491 PMC2636867

[30] Purcell S, Neale B, Todd-Brown K, Thomas L, Ferreira MA, et al (2007) PLINK: a tool set for whole-genome association and population-based linkage analyses. Am J Hum Genet 81: 559–575. 17701901 10.1086/519795 PMC1950838

[31] Price AL, Patterson NJ, Plenge RM, Weinblatt ME, Shadick NA, et al (2006) Principal components analysis corrects for stratification in genome-wide association studies. Nat Genet 38: 904–909. 16862161 10.1038/ng1847

[32] Marchini J, Howie B, Myers S, McVean G, Donnelly P (2007) A new multipoint method for genome-wide association studies by imputation of genotypes. Nat Genet 39: 906–913. 17572673 10.1038/ng2088

[33] Stahl EA, Wegmann D, Trynka G, Gutierrez-Achury J, Do R, et al (2012) Bayesian inference analyses of the polygenic architecture of rheumatoid arthritis. Nat Genet 10.1038/ng.2232 PMC6560362 22446960

[34] Gauthier SA, Glanz BI, Mandel M, Weiner HL (2006) A model for the comprehensive investigation of a chronic autoimmune disease: the multiple sclerosis CLIMB study. Autoimmun Rev 5: 532–536. 17027888 10.1016/j.autrev.2006.02.012

[35] Stegle O, Parts L, Durbin R, Winn J (2010) A Bayesian framework to account for complex non-genetic factors in gene expression levels greatly increases power in eQTL studies. PLoS Comput Biol 6: e1000770 doi:10.1371/journal.pcbi.1000770. 20463871 10.1371/journal.pcbi.1000770 PMC2865505

[36] De Jager PL, Jia X, Wang J, de Bakker PI, Ottoboni L, et al (2009) Meta-analysis of genome scans and replication identify CD6, IRF8 and TNFRSF1A as new multiple sclerosis susceptibility loci. Nat Genet 41: 776–782. 19525953 10.1038/ng.401 PMC2757648

[37] Stranger BE, Nica AC, Forrest MS, Dimas A, Bird CP, et al (2007) Population genomics of human gene expression. Nat Genet 39: 1217–1224. 17873874 10.1038/ng2142 PMC2683249

[38] Parker A, Izmailova ES, Narang J, Badola S, Le T, et al (2007) Peripheral Blood Expression of Nuclear Factor-kappaB-Regulated Genes Is Associated with Rheumatoid Arthritis Disease Activity and Responds Differentially to Anti-Tumor Necrosis Factor-alpha versus Methotrexate. J Rheumatol 34: 1817–1822. 17696278

[39] Batliwalla FM, Baechler EC, Xiao X, Li W, Balasubramanian S, et al (2005) Peripheral blood gene expression profiling in rheumatoid arthritis. Genes Immun 6: 388–397. 15973463 10.1038/sj.gene.6364209

[40] Devlin B, Roeder K, Wasserman L (2001) Genomic control, a new approach to genetic-based association studies. Theor Popul Biol 60: 155–166. 11855950 10.1006/tpbi.2001.1542

[41] de Bakker PI, Ferreira MA, Jia X, Neale BM, Raychaudhuri S, et al (2008) Practical aspects of imputation-driven meta-analysis of genome-wide association studies. Hum Mol Genet 17: R122–128. 18852200 10.1093/hmg/ddn288 PMC2782358

