# Supplementary figures and images for: Genome-Wide Association Study and Gene Expression Analysis Identifies CD84 as a Predictor of Response to Etanercept Therapy in Rheumatoid Arthritis
Source: PLoS Genet. 2013 Mar 28;9(3):e1003394. doi: 10.1371/journal.pgen.1003394 (PMC3610685; doi:10.1371/journal.pgen.1003394)

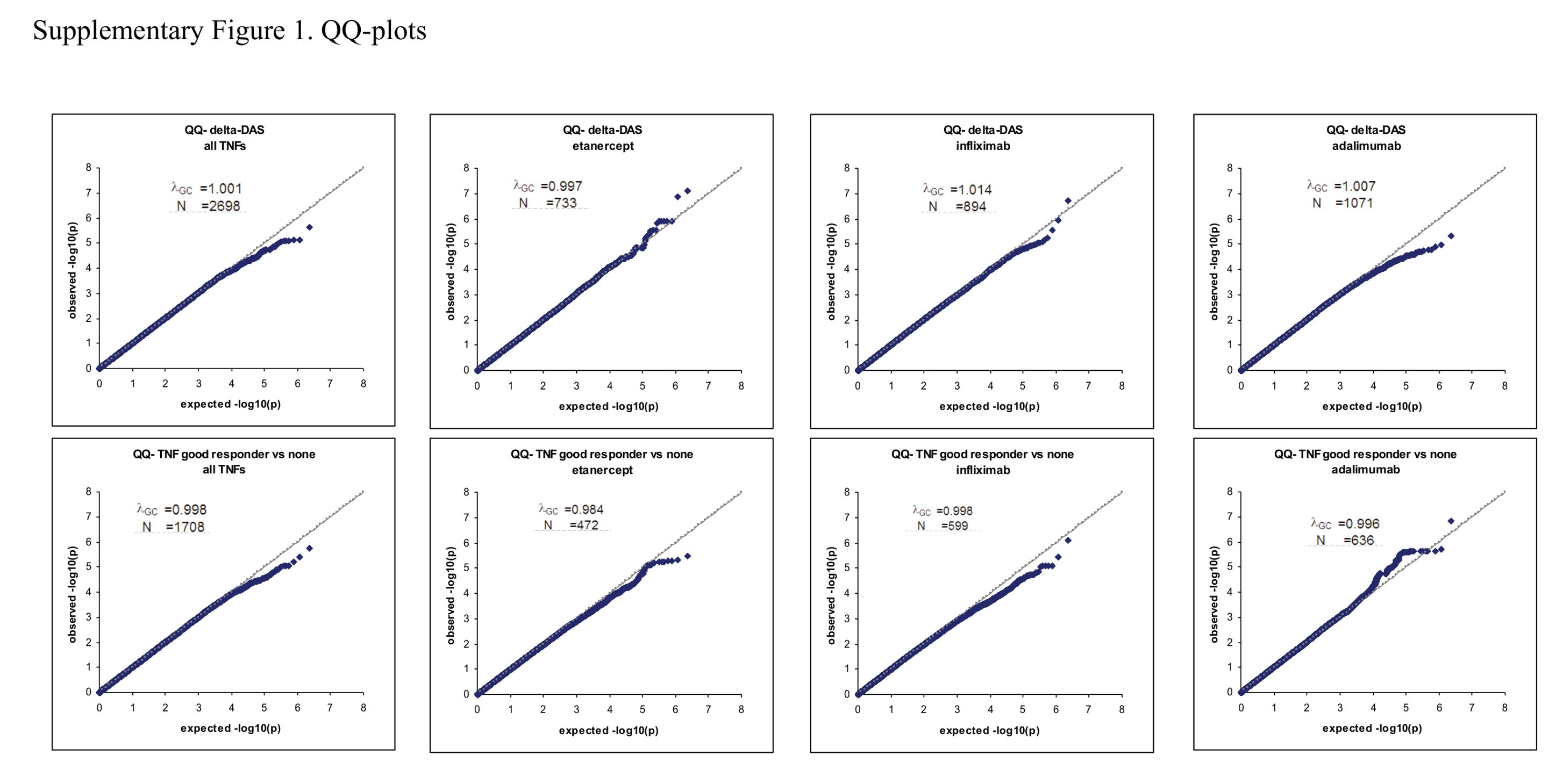

Supplement: Figure S1 — Quantile–quantile (QQ) plots for ΔDAS and response analysis, with genomic control λGC values. (TIF) [file pgen.1003394.s001.tif]

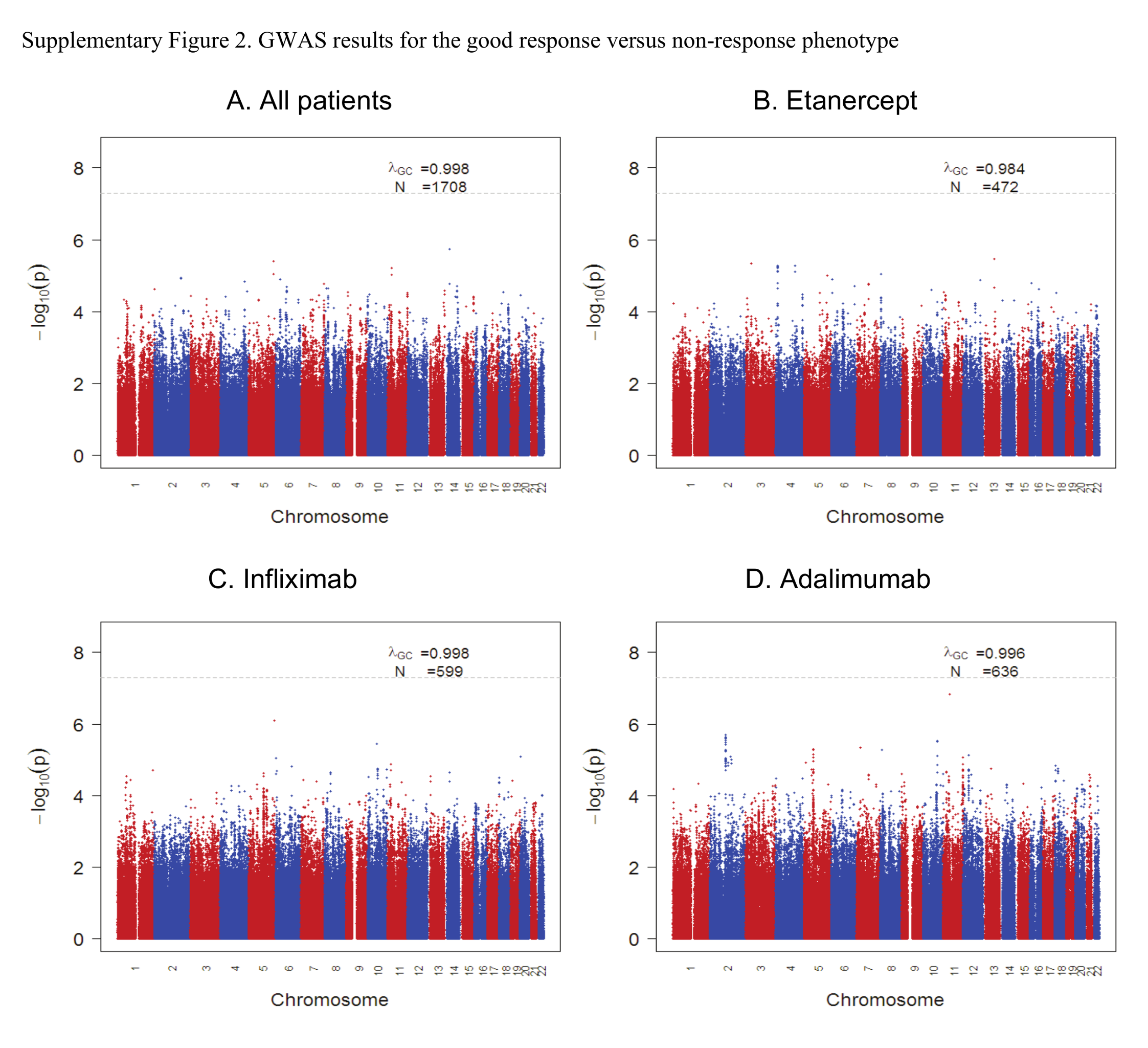

Supplement: Figure S2 — GWAS results for the good response versus non-response phenotype. Shown are strengths of association (−Log10 P-value) for each SNP versus position along chromosomes 1 to 22. A) All samples (n = 1,708). B) Etanercept-treated patients (n = 472). C) Infliximab-treated patients (n = 599). D) Adalimumab-treated patients (n = 636). (TIF) [file pgen.1003394.s002.tif]

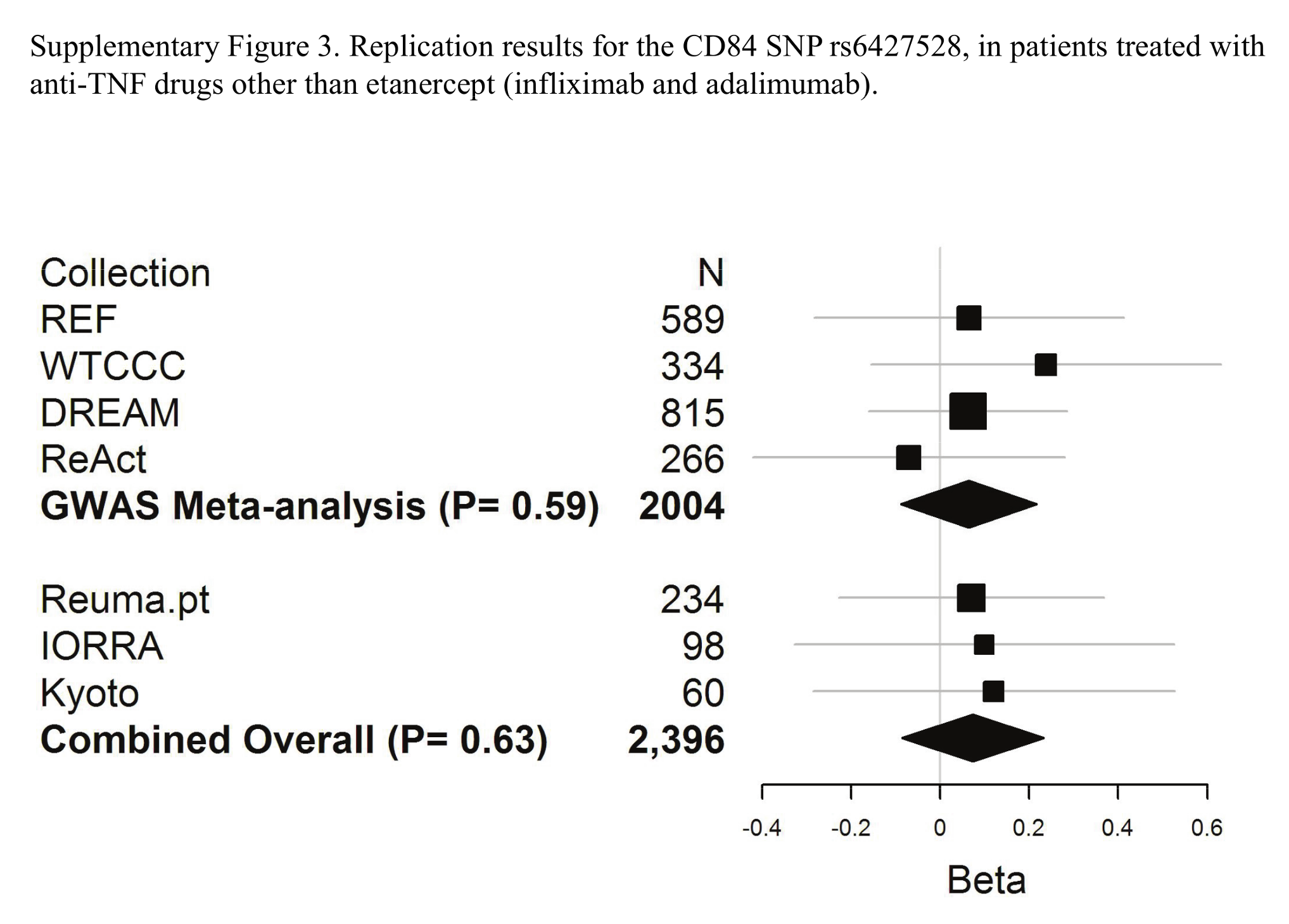

Supplement: Figure S3 — Forest plot of replication results for the CD84 SNP rs6427528, in patients treated with anti-TNF drugs other than etanercept (infliximab & adalimumab). (TIF) [file pgen.1003394.s003.tif]

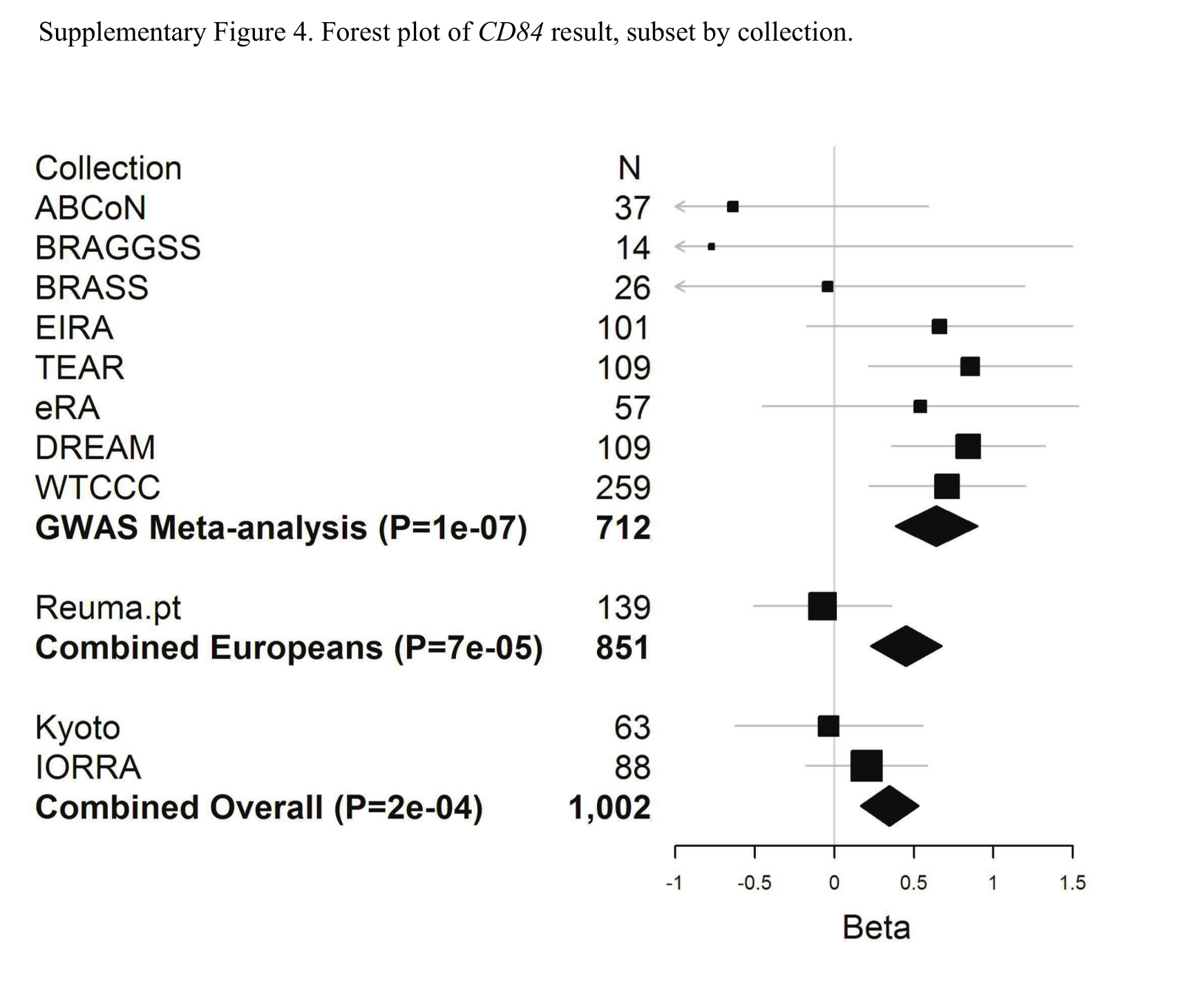

Supplement: Figure S4 — Forest plot of CD84 result in patients treated with etanercept, subset by all collections. (TIF) [file pgen.1003394.s004.tif]

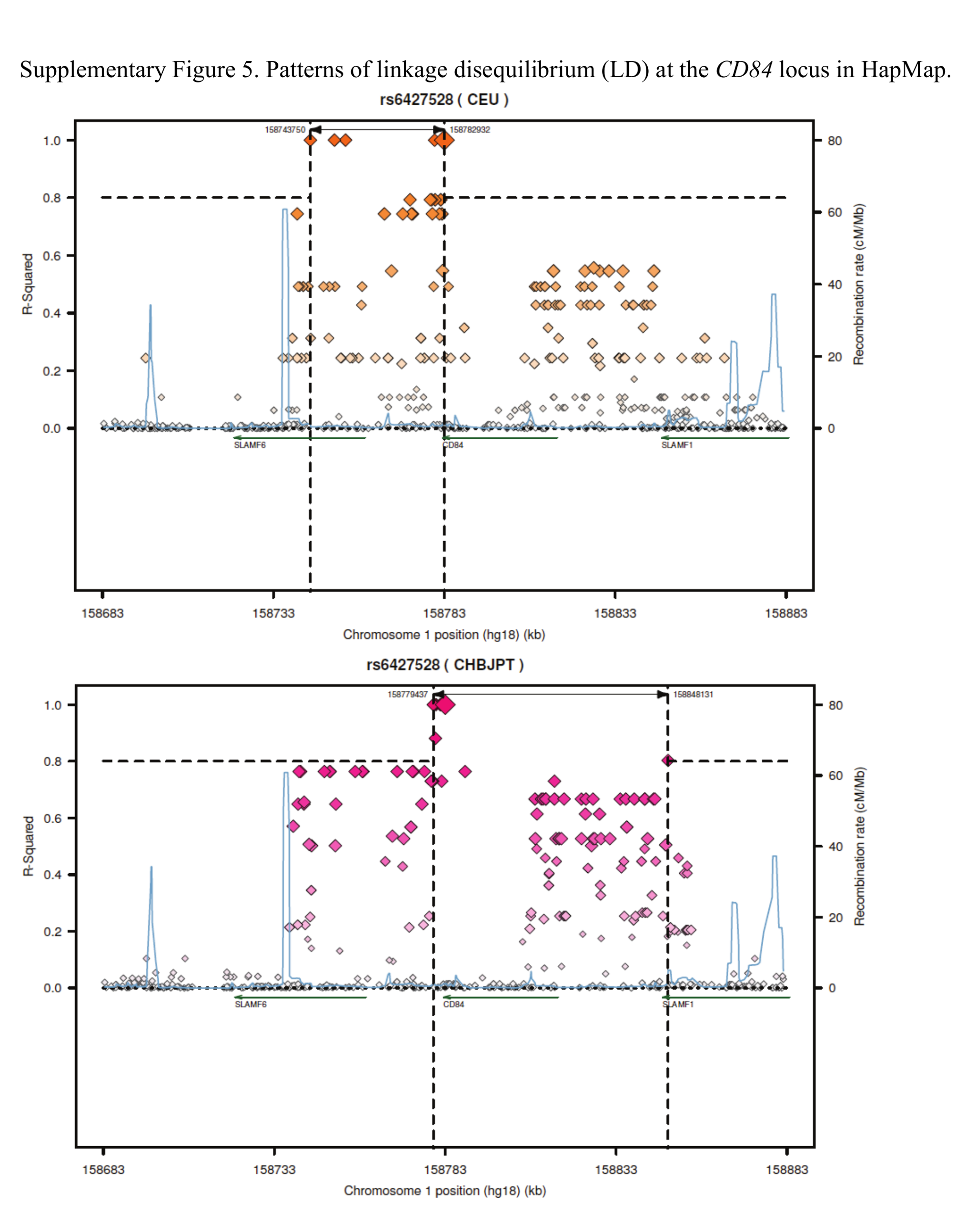

Supplement: Figure S5 — Patterns of linkage disequilibrium (LD) at the CD84 locus in HapMap. Shown patterns of LD for CEU (top panel) and CHBJPT (bottom panel). (TIF) [file pgen.1003394.s005.tif]
