# Supplementary material for: Genome-Wide Association Study and Gene Expression Analysis Identifies CD84 as a Predictor of Response to Etanercept Therapy in Rheumatoid Arthritis
Source: PLoS Genet. 2013 Mar 28;9(3):e1003394. doi: 10.1371/journal.pgen.1003394 (PMC3610685; doi:10.1371/journal.pgen.1003394)
Supplement: Table S1 — Sample information for each of thirteen clinical batches. (DOC) [file pgen.1003394.s006.doc]

Supplementary Table 1. Sample information for each of thirteen clinical batches.

|  | ABCoN | AMC | BeSt | BRAGGSS | BRASS | EIRA | ERA | KI | LUMC | BRAGGSS2 | TEAR | WTCCC | DREAM | ReAct |
| --- | --- | --- | --- | --- | --- | --- | --- | --- | --- | --- | --- | --- | --- | --- |
| design | Prospect | Prospect | RCT | Observ | Observ | Observ | RCT | Observ | Observ | Observ | RCT | Observ | Observ | Observ |
| sample size | 79 | 53 | 86 | 53 | 55 | 299 | 57 | 77 | 47 | 87 | 109 | 592 | 880 | 266 |
| Good Response | 27 | 19 | 62 | 23 | 27 | 112 | 28 | 41 | 2 | 48 | 44 | 160 | 313 | 90 |
| Moderate Response | 33 | 21 |  | 3 | 11 | 106 | 12 | 9 | 1 | 6 | 42 | 257 | 359 | 127 |
| No Response | 19 | 13 | 24 | 27 | 17 | 81 | 17 | 27 | 44 | 33 | 23 | 175 | 208 | 49 |
| drug | all | ADA+IFX | IFX | IFX | all | all | ETN | all | all | all | ETN | all | all | all |
| DAS | DAS28 CRP4 | DAS28 ESR | DAS28 ESR | DAS28 ESR | DAS28 CRP3 | DAS28 ESR | DAS28 CRP4 | DAS28 ESR | DAS28 ESR | DAS28 ESR | DAS28 ESR | DAS28 ESR | DAS28 ESR | DAS28 ESR |
| Genotype Method | Illu 317K | Illu 650K | Illu 650K | Illu 650K | Affy 6.0 | Illu 317K | Illu 550K | Illu 650K | Illu 650K | Illu 650K | Illu 650K | Affy 500K | Illu550K +650K | Illu OmniExpress |
| Time response, mean(range) | 3.3(2.8-15.4) | 3.7(1.9-4.2) | (6-12) | 3.3(2.0-21.4) | (3-12) | 3.4(2.1-5) | 12(10-12.9) | 3.5(2.5-5) | 3.7 | 5.8(2.2-12.2) | 6 | ? | 3 | 3 |
| age, mean(SD) | 54.9(13.4) | 55.3(12.1) | 51.6(14.1) | 55.3(11.3) | 56.6(13.0) | 51.3(12.6) | 51.7(12.9) | 55.9(13.7) | 54.6(11.8) | 55.8(10.8) | 53.6(12.6) | 57.4(10.9) | 54.8(12.9) | 53.9(10.8) |
| sex, Female % | 77.2 | 77.4 | 65.1 | 77.8 | 85.5 | 73.9 | 79 | 81.8 | 58.5 | 81.6 | 72.5 | 77.4 | 68.3 | 78.4 |
| CCP/RF, % | 87.5 | 71.2 | 82.6 | 92 | 100 | 82.6 | 84.2 | 84.4 | 92.9 | 73.9 | 91.7 | 78.2 | 79.7 | 69.9 |
| MTX, % | 68.4 | 92.5 | 96.5 | 90.7 | 25.5 | 74.3 | 0 | 100 | 27.7 | 79.3 | 25.7 | 80 | 76.0 | 50.6 |
| disease duration, mean(SD) | 10.4(9.1) | 12.9(19.6) | 1.4(1.3) | 11.7(9.8) | 9.9(9.8) | 3.2(2.5) | 0.8(0.8) | 13.7(11.5) | 11.4(10.1) | 12.4(9.6) | 2.6(5.2) | 13.9(9.8) | 9.6(9.5) | 11.9(9.1) |
| Start DAS, mean(SD) | 5.1(0.8) | 5.4(1.1) | 5.5(1.0) | 6.7(1.0) | 5.2(1.1) | 5.3(1.3) | 5.0(1.1) | 5.6(1.1) | 3.7(1.4) | 6.2(0.9) | 5.6(1.1) | 6.7(0.9) | 5.5(1.2) | 5.9(1.0) |
