# Supplementary material for: Genome-Wide Association Study and Gene Expression Analysis Identifies CD84 as a Predictor of Response to Etanercept Therapy in Rheumatoid Arthritis
Source: PLoS Genet. 2013 Mar 28;9(3):e1003394. doi: 10.1371/journal.pgen.1003394 (PMC3610685; doi:10.1371/journal.pgen.1003394)
Supplement: Table S2 — Clinical multivariate model for the ΔDAS phenotype. (DOC) [file pgen.1003394.s007.doc]

Supplementary Table 2. Clinical multivariate model for the ΔDAS phenotype.

| Clinical covariate | Beta | SE | P |
| --- | --- | --- | --- |
| age | -0.003 | 0.005 | 0.5 |
| gender | 0.066 | 0.13 | 0.6 |
| mtx1* | -0.29 | 0.13 | 0.03 |
| mtx2** | -0.061 | 0.22 | 0.8 |
| stardas | 0.65 | 0.027 | <.0001 |
| sero | -0.12 | 0.16 | 0.4 |
| Dx duration | -0.0002 | 0.006 | 0.97 |

*mtx1 estimate is not on MTX vs on MTX

**mtx2 estimate is unknown MTX status vs on MTX
