# Supplementary material for: Genome-Wide Association Study and Gene Expression Analysis Identifies CD84 as a Predictor of Response to Etanercept Therapy in Rheumatoid Arthritis
Source: PLoS Genet. 2013 Mar 28;9(3):e1003394. doi: 10.1371/journal.pgen.1003394 (PMC3610685; doi:10.1371/journal.pgen.1003394)
Supplement: Table S4 — Sample and clinical data summary for replication samples. (DOC) [file pgen.1003394.s009.doc]

Table S4. Sample and clinical data summary for replication samples.

| **Collection:** | **Rheuma.Pt** | **IORRA** | **Kyoto** |
| --- | --- | --- | --- |
| **(Ancestry)** | **Portuguese** | **Japanese** | **Japanese** |
| **Sample size** | 405 | 200 | 174 |
| **etanercept** | 153 | 88 | 65 |
| **adalimumab or infliximab** | 252 | 112 | 109 |
| **EULAR Response categories** |  |  |  |
| **Good Responder** | 123 | 74 | 56 |
| **Moderate** | 185 | 75 | 51 |
| **None responder** | 92 | 51 | 38 |
|  |  |  |  |
| **Age, yr; mean (SD)** | 52.0 (12.1) | 54.6 (11.0) | 52.5 (15.1) |
| **Disease duration, yr; mean (SD)** | 10.6 (8.8) | 13.0 (9.2) | 8.8(7.9) |
| **Gender, Female %** | 90.1 | 89.5 | 82.8 |
| **Seropositive, %** | 87 | 97 | 96 |
| **MTX co-therapy, %** | 82.5 | 84.5 | 87.5 |
| **Baseline DAS, mean (SD)** | 5.8 (1.1) | 5.1 (1.2) | 5.0 (1.4) |
| **Delta-DAS, mean (SD)** | 1.8 (1.3) | 1.5 (1.2) | 1.5 (1.2) |
